# The Effect of Uncertainty in Exposure Estimation on the Exposure-Response Relation between 1,3-Butadiene and Leukemia

**DOI:** 10.3390/ijerph6092436

**Published:** 2009-09-11

**Authors:** John J. Graff, Nalini Sathiakumar, Maurizio Macaluso, George Maldonado, Robert Matthews, Elizabeth Delzell

**Affiliations:** 1 Wayne State University School of Medicine, Karmanos Cancer Institute, Detroit, MI 48201, USA; 2 University of Alabama at Birmingham School of Public Health, Department of Epidemiology, Birmingham, AL 35294, USA; E-Mails:nalini@uab.edu (N.S.);rsm@uab.edu (R.M.);edelzell@uab.edu (E.D.); 3 Centers for Disease Control and Prevention, Division of Reproductive Health, Atlanta, GA 30341, USA; E-Mail:mum0@cdc.gov; 4 University of Minnesota School of Public Health, Division of Environmental Health Sciences, Minneapolis, MN 55455, USA; E-Mail:gmphd@umn.edu

**Keywords:** 1,3-butadiene, epidemiology, methods, leukemia, workplace exposures, uncertainty analysis

## Abstract

In a follow-up study of mortality among North American synthetic rubber industry workers, cumulative exposure to 1,3-butadiene was positively associated with leukemia. Problems with historical exposure estimation, however, may have distorted the association. To evaluate the impact of potential inaccuracies in exposure estimation, we conducted uncertainty analyses of the relation between cumulative exposure to butadiene and leukemia. We created the 1,000 sets of butadiene estimates using job-exposure matrices consisting of exposure values that corresponded to randomly selected percentiles of the approximate probability distribution of plant-, work area/job group-, and year specific butadiene ppm. We then analyzed the relation between cumulative exposure to butadiene and leukemia for each of the 1,000 sets of butadiene estimates. In the uncertainty analysis, the point estimate of the RR for the first non zero exposure category (>0–<37.5 ppm-years) was most likely to be about 1.5. The rate ratio for the second exposure category (37.5–<184.7 ppm-years) was most likely to range from 1.5 to 1.8. The RR for category 3 of exposure (184.7–<425.0 ppm-years) was most likely between 2.1 and 3.0. The RR for the highest exposure category (425.0+ ppm-years) was likely to be between 2.9 and 3.7. This range off RR point estimates can best be interpreted as a probability distribution that describes our uncertainty in RR point estimates due to uncertainty in exposure estimation. After considering the complete probability distributions of butadiene exposure estimates, the exposure-response association of butadiene and leukemia was maintained. This exercise was a unique example of how uncertainty analyses can be used to investigate and support an observed measure of effect when occupational exposure estimates are employed in the absence of direct exposure measurements.

## 1. Introduction

Assessment of exposure in epidemiologic studies is especially difficult for historical periods when worker or workplace monitoring data were scarce. Exposure typically must be estimated using information on subjects’ history of employment by production area, job title, task, duration of employment or a combination of these [1,2]. In these situations misclassification of subjects by exposure is inevitable, error in study results is likely and the direction of the error may be unpredictable [3–10]. Uncertainty analysis of exposure estimates can be used to evaluate quantitatively the impact of exposure measurement error on study results and can improve the interpretation of study results [11–13]. Uncertainty analysis is an approach in which the statistical analysis is systematically repeated, using different assumptions each time. This technique may be used to measure how sensitive results are to changes in assumptions about selection bias, information bias and confounding. In an uncertainty analysis, one may repeat the analysis using different corrections for possible unintentional omissions from the eligible study group for misclassification of subjects by exposure or outcome or for uncontrolled confounding [14–17].

In a retrospective follow-up study of mortality among North American synthetic rubber industry workers, cumulative exposure to 1,3-butadiene (butadiene), a carcinogen [18] of substantial industrial importance [19], was positively associated with leukemia. Evidence of exposure-response persisted after controlling for potential confounding by other agents [20]. Problems with historical exposure estimation, however, may have distorted the observed association between butadiene and leukemia.

Here we report the results of an uncertainty analysis that examines quantitatively the impact of potential inaccuracies in exposure estimation on the observed results of an investigation of the relation between cumulative exposure to butadiene and leukemia.

## 2. Methods

### 2.1. Overview of Exposure Estimation

The approach used to estimate exposure in our study of synthetic rubber workers has been described previously [21,22]. Development of exposure estimates did not use industrial hygiene data for several reasons. There have been extensive changes in production processes and engineering controls in the synthetic rubber industry since it began in the 1940s, historical exposure measurements were sparse before 1975, and exposure measurements taken since 1975 did not cover all work area/job groups at all plants and may have underestimated butadiene concentrations.

Estimation procedures, explained in detail elsewhere [21,22], included: 1) identifying at each plant a series of work area/job groups that had similar job tasks and exposure potential, 2) identifying for each work area/job group its component tasks that entailed exposure, documenting historical changes in those tasks, and estimating exposure time and intensity (parts per million, ppm) associated with each task; 3) calculating calendar time period-specific eight-hour time-weighted average (TWA) exposure intensities for each work area/job group and compiling these into job-exposure matrices (JEMs), and 4) linking the exposure estimates in the JEMs with each subject’s work history to obtain cumulative exposure estimates. To better illustrate the exposure estimation process used in our main analyses and the approximate probability distributions associated with the butadiene exposure estimates, we have included an Appendix that outlines one subject’s work history, with its calendar year and work area/job group combinations and the corresponding calculated butadiene estimates.

To develop information on exposure and on historical changes in exposure potential for each of the work area/job groups, we conducted in-depth walk-through surveys at each of the six plants, met with knowledgeable plant staff, obtained engineering and construction records, and interviewed workers who had a history of long-term employment in specific work area/job title groups. The interviews provided information on process layout, equipment and material flow, process operations, job titles of workers employed in routine operations or maintenance/cleanup, potential exposure sources and exposure control systems.

We developed an integrated system of computer programs to assist with documenting and calculating exposure estimates. This system consisted of an interactive Statistical Analysis System-AF [23] interface that integrated text descriptions of each task and information on the exposure scenario, on the exposure estimation assumptions and on calculations documenting the exposure estimates for each task and time period. The menu driven interface enabled investigators to review and modify estimation assumptions (i.e., probability of an operator standing in the emission plume, wind speed, frequency and duration of task, and distance of operator from point source of emission), to recalculate task and/or work area/job group-specific estimates and to obtain the approximate probability distribution of butadiene intensity (ppm) for each combination of plant, work area/job group and calendar year. The end result was a JEM, each cell of which contains a distribution of butadiene ppm estimates. The Appendix (Illustration of Exposure Estimation) further summarizes exposure estimation procedures by illustrating the derivation of the approximate probability distribution of butadiene ppm estimates for one plant/task/year combination and the use of these estimates in obtaining the corresponding distribution for a plant/work area/job group/year combination.

### 2.2. Task-Specific Exposure Estimates

We used information obtained from interviews with plant hourly and salaried staff and direct observation of the work place to compute a distribution of exposure estimates for each task in each time period during which exposure determinants could be considered constant. We then compiled task-specific exposure estimates into a task-exposure matrix and identified the tasks comprising each work area/job group.

In brief, we derived each time period -specific distribution of estimates for each task by specifying a lower and upper boundary for the parameters in an exposure model with the following parameters: emission rate, ventilation rate/air speed, and, when appropriate, the distance of the worker from a point source of emissions for each task entailing exposure other than background (see Appendix, section II). We assumed that each parameter in the exposure model followed a triangular distribution with the mode at the midpoint between the boundaries. We then used simulation to compute an approximate probability distribution of the butadiene exposure intensity for a task. We further computed the approximate distribution of the sum over one shift of butadiene ppm-minutes associated with a particular task by assuming that the task’s duration and frequency followed a triangular distribution. We evaluated the resulting empirical distributions to find the approximate 1^st^, …, 99^th^ percentile of each task- and time period-specific exposure intensity estimate.

### 2.3. Work Area/Job Group Exposure Estimates

To obtain eight-hour TWA estimates for each work area/job group, we developed algorithms to combine task-specific estimates with background estimates (Appendix, Section III). These algorithms first multiplied the intensity for each task by the task-specific minutes of exposure occurring during a work shift to obtain the task-specific number of ppm-minutes; next, multiplied the remaining part of the time period of the shift by the estimated area background intensity to obtain the number of ppm-minutes of background exposure; and last, divided the sum of ppm-minutes of exposure by 480 to obtain the eight-hour TWA in ppm. Thus, the algorithms considered for each task comprising the work area/job group, the frequency and duration of the task during an eight-hour work shift.

### 2.4. Subject-Specific Cumulative Exposure Estimates

We linked exposure estimates for each work area/job group with the work histories of individual workers and computed final lifetime cumulative exposure indices. The latter computation involved multiplying the calendar year-specific amount of time a worker spent in each work area/job group by the concentration (ppm) or annual number of peaks estimated for that work area/job group and calendar year category, and summing over all work area/job title groups and years covered by a subject’s employment history.

### 2.5. Vital Status and Cause of Death Information

We used linkages with several national databases, including the National Death Index (NDI), Social Security Administration (SSA) and Canadian Mortality Database (CMDB), to update vital status of the study group. Cause of death information came from a combination of death certificate information (for subjects who died before 1979, the beginning of the NDI) and linkages to NDI *Plus* and the CMDB. We attempted to obtain medical records for all subjects whose death certificate mentioned leukemia. For analyses, we included as leukemia decedents those subjects whose medical records confirmed a diagnosis of leukemia and subjects whose death certificate indicated an underlying or contributing cause of death from leukemia.

### 2.6. Association of Butadiene Exposure to Leukemia Mortality in the Main Analysis

Poisson regression analyses of the relation between cumulative exposure to butadiene and leukemia mortality among the study group included 500,174 person-years of observation and 81 decedents with leukemia. Evidence of exposure-response persisted, after controlling for age, years since hire and potential confounding by other agents [20].

### 2.7. Uncertainty Analyses

Uncertainty analyses evaluated the impact of potential inaccuracies in butadiene exposure estimation on relative rates (RRs) for leukemia. In these analyses we examined subjects’ leukemia mortality rates in relation to each of 1,000 sets of butadiene cumulative exposure (ppm-years) estimates.

To obtain the *i*th (*i*, 1–1000) set of exposure estimates for a particular plant, for each work area/job group at that plant we: 1) randomly selected a percentile (1–99%) and used the exposure estimate corresponding to this percentile for each work area/job group, 2) obtained, from each year-specific approximate probability distribution of exposure estimates for that work area/job group, the butadiene ppm value corresponding to the selected percentile, 3) repeated percentile selection for each work area/job group 1000 times, and 4) compiled the complete work area/job group-year butadiene ppm JEM for the *i*th iteration. After we combined the butadiene ppm values selected for each plant during the *i*th iteration of the procedure to obtain JEM (*i*), we linked JEM (*i*) to work history data to obtain the *i*th set of butadiene ppm-years for each subject. We then analyzed the relation between cumulative exposure to butadiene and leukemia for the data derived from each of the 1,000 JEMs (Figure 1).

Poisson regression analyses used the Statistical Analysis System GENMOD procedure [23] to obtain maximum likelihood estimates of leukemia RRs for categories of butadiene ppm-years (>0–<33.7, 33.7–<184.7, 184.7–<425.0, and 425.0+ ppm-years), controlling for age and years since hire. We specified exposure categories based on the distribution of cumulative butadiene exposure among leukemia decedents, using quartiles of cumulative exposure among those decedents with nonzero exposure.

### 2.8. Exposure-Response Simulation

To assess any observed exposure-response association of butadiene ppm-years and leukemia, we performed a simulation that determined how often we would see a monotonic increase in RRs in the four exposure categories, due to chance alone. Using data from the main analysis [20], we determined the proportional distribution of leukemia cases that would yield all RRs = 1.0 after adjusting for covariates. Briefly, we took the observed value for each exposure category and divided it by the adjusted RR to compute the theoretical number of cases that would yield RR = 1.0. Next, we summed all expected values and recalculated the proportion of the total accounted for by each expected value. We, then used the set of values calculated above as the parameters of a multinomial distribution with N = 81 (the total number of leukemia cases observed), and generated first 10, then 1,000, then 10,000 and finally 1,000,000 samples of size 81, which represented a control population for the 1,000 alternative cohorts in the uncertainty analyses. For each sample, we counted the number of contrasts between adjacent RRs that were consistent with a positive or negative exposure-response.

## 3. Results

As expected, percentiles chosen for each primary work area/job group in the six plants ranged from a minimum value of 1 to a maximum of 99. Median values for the selected percentiles ranged from 43.5 for one work area/job group in plant seven to 54.0 for three work area/job groups in plant seven and one work area/job group in plant one. Among the 1,000 datasets, minimum and maximum selected percentiles ranged from 1 to 20 and from 83 to 99, respectively. Median percentiles ranged 27.5–73.0, and the arithmetic mean of selected percentiles ranged from 35.0 to 67.2 (data not displayed).

Leukemia RRs for the lowest nonzero category of butadiene ppm-years (category 1) ranged from a minimum of 1.2 to a maximum of 1.8 (Table 1). In categories 2, 3, and 4 of butadiene ppm-years, the ranges of RRs were 1.1–2.2, 1.2–3.8, and 2.4–4.3, respectively. The RR median values indicated a positive association between butadiene ppm-years and leukemia with RRs of 1.0, 1.5, 1.6, 2.6, and 3.3, respectively, for exposures of 0, >0–<33.7, 33.7–<184.7, 184.7–<425.0, and 425.0 + ppm-years.

Among the 1,000 uncertainty analysis datasets, 473 indicated a regular exposure-response relation between butadiene ppm-years and leukemia (data not presented), in that the RR from each nonzero category of butadiene ppm-years was greater than the RR for the next lower category. Among the 473 datasets that indicated a regular exposure-response pattern, the median change in RR between adjacent exposure categories was 20% for categories 1 and 2, 41% for categories 2 and 3, and 35% for categories 3 and 4. In the absence of a control distribution reflecting the null hypothesis of no association, we undertook an exercise to determine how often we would see a monotonic increase in RRs due to chance alone. Under the null hypothesis, only five or six of the 1,000 uncertainty analyses should have resulted in a monotonically increasing risk level within the four exposure categories, while we observed 473, even after perturbing the exposure estimates throughout the ranges of uncertainty that we designed.

**Figure 2.**
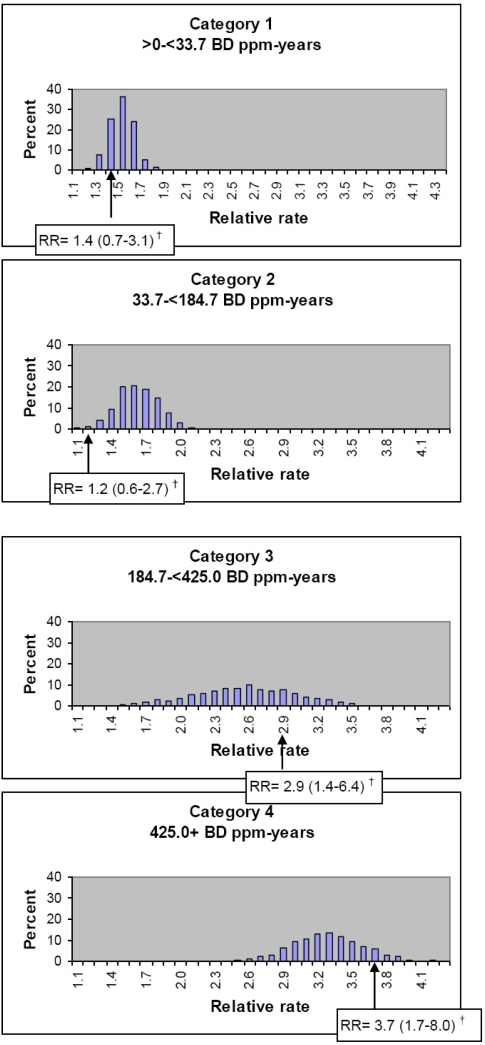
Distribution of leukemia relative rate by category of butadiene ppm-years*, 1,000 uncertainty analysis Poisson regression models. * Categories were quartiles of BD ppm-years among exposed leukemia decedents, used in main analyses, in which exposure estimates were derived from a job-exposure matrix containing eight-hour time weighted average butadiene intensities corresponding to the mean of the approximate probability distribution of estimates for each plant/work area/job group/calendar year combination. ^†^ Leukemia relative rate and 95% CI for BD ppm-years in the main analysis.

In exposure category 1 of butadiene ppm-years, 25% of the RRs had a value of 1.4, the same value as in the main analysis (Figure 2). Eight percent of the RRs in category 1 were less than 1.4, and 67% of the RRs were greater than 1.4. In exposure category 2, only 1% of the RRs had the same value as in the main analysis (RR = 1.2). Almost all (99%) of the RRs in exposure category 2 were greater than 1.2, whereas less than 1% had a value lower than 1.2. In both categories 3 and 4, the majority of RRs were less than the corresponding RR from the main analysis.

In 166 (31%) of the 527 datasets that did not display a monotonic exposure-response pattern, the lack of montonicity was due to the fact that two or more adjacent exposure categories had the same RR (Table 2). In 79 the RR from exposure category 4 was less than the RR from category 3. In 44 the RR from category 3 was less than the RR from category 2. In 199 the RR from category 2 was less than the RR from category 1. In 38 the RR from category 4 was less than the RR from category 3, and the RR from category 2 was less than the RR from category 1.

Figure 2 can be interpreted as a probability distribution that describes our uncertainty in RR point estimates due to uncertainty in exposure estimation. Figure 2 shows that, under our assumptions in the uncertainty analysis about the exposure estimation parameters and under our analysis assumptions, the point estimate of the RR for category 1 of butadiene ppm-years is most likely to be about 1.5; it is unlikely to be less than 1.2 or greater than 1.9. The RR for category 2 is most likely to be about 1.5–1.8; it is unlikely to be less than 1.1 or greater than 2.0. The RR for category 3 is most likely to be about 2.1–3.0; it is unlikely to be less than 1.5 or greater than 3.4. The RR for category 4 is likely to be about 2.9–3.7; it is unlikely to be less than 2.5 or greater than 4.2.

## 4. Discussion

In most epidemiologic research, the amount of error in a measure of effect is presented in a confidence interval, which is simply an indication of random error or the effect measure’s precision. However, the amount of error due to the effect measure’s validity, the systematic error, rarely is presented. A quantitative assessment of the systematic error for an effect estimate can be made by conducting uncertainty analysis [14–17,24–29].

In our study of mortality among North American synthetic rubber industry workers, we assessed the impact of potential systematic error due to problems with historical exposure estimation on the observed association between butadiene and leukemia. When comparing the distribution of RRs from the uncertainty analyses to those in our main analysis, in which the exposure estimates were derived from a JEM containing butadiene intensities corresponding to the mean of the approximate probability distribution of estimates for each plant/work area/job group/calendar year combination, the main analysis RRs in the first two exposure categories fell at the low end of the distribution of RRs from the uncertainty analyses and were at the high end of the distribution in the third and fourth exposure categories. Nonetheless, after considering the complete probability distributions of butadiene exposure estimates, the exposure-response association of butadiene and leukemia is maintained.

There are alternatives to the procedures we used to assess uncertainty stemming from exposure estimation. One possible approach could include the arbitrary variation of assumptions made about TWA estimated exposure for particular work area/job groups. The amount of misclassification of exposure most likely varies among work area/job group estimates. Misclassification may be greatest for work area/job groups that are “nonspecific” (i.e., Production Operator, Production Laborer, Maintenance Laborer or Laboratory worker in unspecified work areas). It is reasonable to assume that a relatively large amount of error occurred in assigning exposure estimates to subjects’ person-time in these groups. Uncertainty analyses could assess how important the lack of job title specificity is in adding to the uncertainty of exposure estimation.

In a preliminary set of uncertainty analyses (data not presented), we created a series of alternative exposure profiles, focusing on work area/job groups that were poorly specified, to evaluate the effect of changes in exposure estimation criteria on the association of butadiene ppm-years and leukemia. We assigned each work area/job group to one of four major categories: unskilled labor in maintenance, skilled trades/field assignment, laboratory technicians and other jobs. We then assigned one of three values of the probability distribution of butadiene estimates (5^th^ percentile, mean or 95^th^ percentile) to each of the four work area/job group categories. The analysis included 10 different exposure profiles and indicated that assumptions made in exposure estimation had little impact on the relation between cumulative butadiene exposure and leukemia. The exposure-response association of butadiene with leukemia persisted in analyses of all 10 exposure profiles. However, this crude analysis had a potential problem in that bias due to exposure estimation error is a complicated function of several parameters, and therefore, examining these few scenarios did not capture the true range of possible estimation error bias.

Using our automated exposure estimation system, we were able to create a much broader range of exposure profiles by creating 1,000 JEMs and subsequently preparing 1,000 datasets for the analysis of the association between butadiene ppm-years and leukemia. The resulting set of RRs portrayed a probability distribution of the estimated RR of the butadiene-leukemia association. These uncertainty analyses assessed the global impact of uncertainty due to exposure estimation on the butadieneleukemia association. The approach entailed manipulation of estimated exposure by using JEMs consisting of exposure values that corresponded to randomly selected percentiles of the approximate probability distribution of plant-, work area/job group- and year-specific butadiene ppm.

This approach was limited in that we were not able to identify particular assumptions (i.e., wind speed, distance of operator from point source of emission, probability of operator standing directly in the emission plume, exposure frequency and duration) that contributed the greatest amount of uncertainty to butadiene exposure estimation. We were also limited to selecting percentile values of butadiene ppm from year-specific approximate probability distributions of exposure estimates for well-defined primary work area/job groups. Butadiene estimates for the less well-defined secondary job groups were, in turn, computed after the percentile estimates were selected for primary work area/job groups. Therefore, this analysis directly quantifies only the variability in the butadiene-leukemia association due to uncertainty in the estimation of butadiene in primary work area/job groups.

This uncertainty analysis was designed to provide insight into the impact of limitations due to exposure estimation procedures, but was carried out only for butadiene ppm-years and leukemia. We estimated exposure for two additional agents in our synthetic rubber workers study, styrene and sodium dimethyldithiocarbamate (DMDTC). Additional analyses could use the same techniques outlined above to investigate the effect on leukemia mortality of uncertainty in styrene and DMDTC exposure estimation.

While additional investigations of the effect of uncertainty in our exposure estimation procedures could be performed, this exercise was a unique approach that displayed the possible distortion of the association observed in our main analysis between cumulative exposure to butadiene and leukemia.

Few occupational and environmental epidemiologic studies have made an effort to quantify the amount of systematic error introduced when using quantitative exposure estimates. This exercise is an example of how uncertainty analyses can be used to investigate and support an observed measure of effect when occupational exposure estimates are employed in the absence of direct exposure measurements.

## Figures and Tables

**Figure 1.**
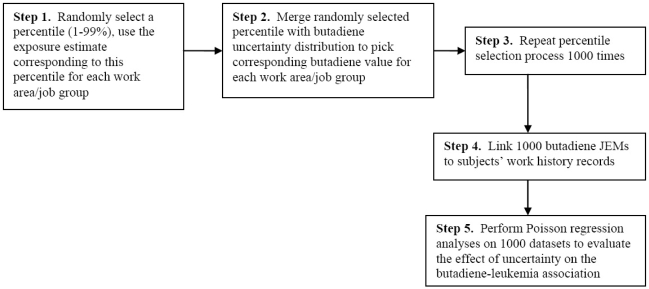
Creation of 1,000 datasets for uncertainty analyses.

**Table 1.** Summary of relative rates for butadiene ppm-years and leukemia from uncertainty analyses of 1,000 alternative datasets of exposure estimates.

Butadiene ppm-years*	**Relative rate**			
	**Minimum**	**Maximum**	**Mean**	**Median**

0	1.0	1.0	1.0	1.0
>0–<33.7	1.2	1.8	1.5	1.5
33.7–<184.7	1.1	2.2	1.6	1.6
184.7–<425.0	1.2	3.8	2.6	2.6
425.0+	2.4	4.3	3.3	3.3

* Categories based on quartiles of exposed leukemia decedents, used in main analyses, in which exposure estimates derived from a job-exposure matrix containing eight-hour time-weighted average butadiene intensities that corresponded to the mean of the approximate probability distribution of estimates for each plant/work area/job group/calendar year combination.

**Table 2.** Number of datasets displaying an nonmonotonic dose-response pattern by type of pattern.

Nonmonotonic pattern*	**N**

RR_2_ = RR_1_	116
RR_3_ = RR_2_	19
RR_3_ = RR_2_ = RR_1_	1
RR_4_ = RR_3_	20
RR_4_ = RR_3_ & RR_2_ = RR_1_	10
RR_4_ < RR_3_	59
RR_4_ < RR_3_ & RR_2_ = RR_1_	20
RR_3_ < RR_2_	43
RR_3_ < RR_2_ = RR_1_	1
RR_2_ < RR_1_	182
RR_4_ = RR_3_ & RR_2_ < RR_1_	17
RR_4_ < RR_3_ & RR_2_ < RR_1_	38
RR_3_ < RR_2_ < RR_1_	1

* RR_1_, relative rate for >0–<33.7 butadiene ppm-years; RR_2_, relative rate for 33.7–<184.7 butadiene ppm-years; RR_3_, relative rate for 184.7–<425.1 butadiene ppm-years; RR_4_, relative rate for 425+ butadiene ppm-years.

**Figure 1A.**
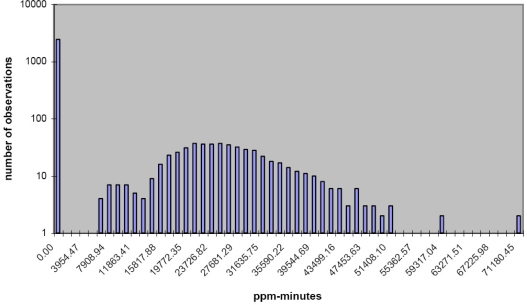
Distribution of butadiene ppm-minutes task 305, plant 4, 1943–1983.

## Appendix

### Illustration of Exposure Estimation

#### Introduction

To illustrate exposure estimation, we selected one subject’s work history, with its calendar year and work area/job group combinations and the corresponding butadiene estimates used in our main analyses. We then selected one combination of work area/job group/calendar year combination from the subject’s work history, described the derivation of butadiene estimates for one of the tasks comprising that work area/job group/year, and calculated the butadiene estimates for the selected work area/job group/year using data from all of its component tasks. Finally, we summarized the derivation of the subject’s butadiene cumulative exposure estimates for one of the uncertainty analysis datasets.

#### I. Overview of One Subject’s Work History and Butadiene Cumulative Exposure Estimates

The subject whom we selected worked at plant four for 28 years, and his work history consisted of 38 segments, each generated at the end of a calendar year or at the point when the subject changed work area/job group during a calendar year (Table 1A). Each segment of the work history included data on the start and end dates of the segment, the work area/job group, the number of days in the segment, the estimated eight-hour time weighted average (TWA) butadiene exposure intensity in parts per million (ppm), and the estimated cumulative butadiene exposure (ppm-years) as of the end of that segment. The butadiene ppm estimates displayed in Table 1A came from the JEM used in the main analysis. That is, each corresponded to the mean of the approximate probability distribution of butadiene intensity (ppm) estimates for the plant/work area/job group/year combination. The subject’s cumulative butadiene exposure was ppm-years.

**Table 1A.** Work history of one subject with butadiene (BD) ppm used in main analyses.

**Segment**	**Work area/job group**	**Start date**	**End date**	**Days**	**BD ppm 8-hour TWA**	**BD ppm-years**
1	812	08/27/1943	09/24/1943	28	36.0066	2.76
2	812	09/24/1943	12/31/1943	98	36.0066	12.42
3	812	12/31/1943	01/23/1944	23	36.2725	14.71
4	812	01/23/1944	10/01/1944	252	36.2725	39.73
5	817	10/01/1944	12/31/1944	91	43.1242	50.48
6	816	12/31/1944	12/12/1945	346	42.9674	91.18
7	816	12/12/1945	12/31/1945	19	42.9674	93.41
8	816	12/31/1945	01/06/1946	6	42.5815	94.11
9	816	01/06/1946	12/31/1946	359	42.5815	135.97
10	816	12/31/1946	12/31/1947	365	42.7788	178.72
11	816	12/31/1947	10/31/1948	305	37.3081	209.87
12	817	10/31/1948	12/31/1948	61	43.1242	217.07
13	817	12/31/1948	12/31/1949	365	43.1242	260.17
14	817	12/31/1949	10/22/1950	295	43.1242	295.00
15	817	10/22/1950	12/31/1950	70	43.1242	303.26
16	817	12/31/1950	12/31/1951	365	43.1242	346.36
17	817	12/31/1951	12/31/1952	366	43.1242	389.57
18	817	12/31/1952	12/31/1953	365	43.1242	432.66
19	817	12/31/1953	12/31/1954	365	43.1242	475.76
20	817	12/31/1954	12/31/1955	365	43.1242	518.85
21	817	12/31/1955	12/31/1956	366	43.1242	562.06
22	817	12/31/1956	12/31/1957	365	43.1242	605.16
23	817	12/31/1957	12/31/1958	365	43.1242	648.25
24	817	12/31/1958	12/31/1959	365	43.1242	691.35
25	817	12/31/1959	12/31/1960	366	40.4189	731.85
26	817	12/31/1960	12/31/1961	365	40.4189	772.24
27	817	12/31/1961	12/31/1962	365	40.4189	812.63
28	817	12/31/1962	03/14/1963	74	40.4189	820.82
29	817	04/30/1963	12/31/1963	245	40.4189	847.93
30	817	12/31/1963	12/31/1964	366	40.4189	888.44
31	817	12/31/1964	12/31/1965	365	40.4189	928.83
32	817	12/31/1965	12/31/1966	365	40.4189	969.22
33	817	12/31/1966	12/31/1967	365	40.4189	1009.61
34	817	12/31/1967	12/31/1968	366	40.4189	1050.11
35	817	12/31/1968	10/31/1969	304	40.4189	1083.75
36	817	10/31/1969	12/31/1969	61	40.4189	1090.50
37	817	12/31/1969	12/31/1970	365	40.4189	1130.89
38	817	12/31/1970	04/29/1971	120	40.4189	1144.17

* Work area/job group 812 = polymerization operative, unspecified; 816 = Polymerization reactor recovery; 817 = recovery in SBR polymerization.

#### II. Derivation of Butadiene Estimate for One of the Tasks Comprising One Work Area/Job Group

Work area/job group 817 (recovery in SBR polymerization, including compressor house and high solids recovery operations), in which the subject worked from 1948 through 1971, consisted of five tasks that entailed exposure to butadiene (Table 2A). We selected task 305 (minor maintenance, recovery compressor house) to illustrate the estimation of task-specific butadiene exposure intensities.

**Table 2A.** Component tasks entailing butadiene exposure, work area/job group 817 (recovery in SBR polymerization, including compressor house and high solids recovery operations), plant 4.

**Task number**	**Task name**

301	Recovery area background
303	Water draw-off from vacuum pumps
305	Minor maintenance of recovery compressor house
312	Drain water from butadiene decanter (recycle tanks)
315	Minor maintenance of butadiene pumps

Table 3A describes task 305 and indicates the exposure scenario and the parameters used to calculate the distribution of butadiene intensity estimates for the task for the time period 1943–1983. There was no butadiene exposure in task 305 after 1983 at plant four.

**Table 3A.** Description of task 305, exposure scenario, and parameters used to estimate exposure to butadiene.

**Description** The inspection and maintenance of the recovery compressors involved inspecting the area for compressor leaks and preparing the compressor for repair by a mechanic or pipefitter. Exposure is a function of the compressor leak rate. The leak rate for compressors was determined to be 20–30 lbs per day. The compressors leaked liquid that was approximately 90% butadiene. The average wind speed values (lower and upper limit) for this task across all plants was used. We assumed that, during the inspection, the operator maintained an average distance of 1 meter from any one of the four compressor seals. The upper and lower limits were calculated based on the theory that the probability that the operator stood directly in the plume was 0.125 (lower limit) to 0.25 (upper limit) of standing directly in the plume. **Exposure scenario** Point source emission of butadiene. During the time period 1943–1983, compressors leaked a water/butadiene mixture at a rate of 20–30 lbs/day; 90% of this mixture was butadiene; thus 18–27 lbs of butadiene were lost from each seal per compressor per day.	
**Parameters**	
• Butadiene emission rate, Q	
– Lower limit = 18 lb/day * 454 grams/1lb * 1day/24 hours * 1 hour/3600 seconds = 0.09458 g/sec – Upper limit = 27 lb/day * 454 grams/1lb * 1day/24 hours * 1 hour/3600 seconds = 0.141875 g/sec	
• Duration of task (minutes)	
– Lower limit = 10 – Upper limit = 20	
• Duration of exposure	
– Lower limit = 10 – Upper limit = 20	
• Frequency of task = 4 times/shift	
• Distance of the operator = 1 meter	
• Wind speed (meters/second)*	
– Upper limit = 0.42 – Lower limit = 1.44	
• Probability of operator standing directly in the plume	
– Lower limit = 0.125 – Upper limit = 0.25	

* Upper limit is lower than lower limit because the lower the wind speed, the higher the exposure.

Butadiene exposure in task 305 occurred if an operator stood in the plume generated by leaking compressors (exposure scenario, point source of emissions). The probability of nonzero exposure for an operator in task 305 (i.e., the probability that an operator stood in the emission plume) ranged from 0.125 to 0.25. Because the maximum probability of exposure was low, many of the probability distribution’s exposure estimates had a value of zero (Figure 1A). The task had seven other exposure parameters, relevant to calculating estimates under nonzero exposure conditions. Six of these could vary in value, and one (distance of the operator from the emission source) had only one value. We used the point source emissions exposure scenario and the parameter estimates listed in Table 3A to estimate the distribution of nonzero values of the butadiene partial TWA for task 305, as shown in Table 4A, and obtained a lower limit of 18.1886 ppm and an upper limit of 187.0889 ppm. The full dataset of estimates consisted of 3,000 observations. From the complete approximate probability distribution, we selected 99 percentile values of the butadiene exposure intensity estimates for task 305 (Table 5A). The first 80 percentiles had a butadiene exposure intensity of zero because of the high probability of zero exposure in task 305.

**Table 4A.** Exposure estimation model and calculation of lower and upper limits of nonzero butadiene partial TWA, task 305.

**Description** Intensity of exposure originating from a point source was calculated using a near-field air dispersion model that estimates worker exposure to gases and vapors leaking from pumps and valves: ${\text{E}}_{\text{ppm}}\;=\;1000\;*\;24.45\;*\;\text{Q}/(\text{MW}\;*\;0.136\;*\;{\text{D}}^{1.84}\;*\;\text{u})$ where E_ppm_ was the estimated air concentration in ppm of butadiene in the plume originating from the emission source, Q was the butadiene emission rate (lower limit = 0.09458; upper limit = 0.141875 grams/second), MW was the molecular weight of butadiene (54.1), D was the distance of the operator from the emission source (1 meter), and u was the air speed across the dispersion field (lower limit = 1.44, upper limit = 0.42 meters/second). The constants in the denominator of the model are dispersion coefficients from the Gaussian model for predicting downwind concentrations due to dispersion of a gas or vapor. Information from interviews indicated that duration of task 305 ranged from a lower limit of 10 minutes to an upper limit of 20 minutes, with an exposure frequency of four times per shift. The partial eight-hour time weighted average was calculated as the point source exposure (E_ppm_) multiplied by the duration and frequency of the task, divided by 480 (the number of minutes in an eight-hour shift). The probability of an operator standing directly in the plume and having an exposure greater than zero ranged from a lower limit of 0.125 to an upper limit of 0.25. If the operator was not directly in the plume, exposure would have been equal to zero. Therefore, the majority of exposure estimates for task 305 had a value of zero (see Figure B1).	
**Calculation of nonzero exposure values**	
Lower limit (LL):	
Point source exposure, if operator was in the emission plume:	E_ppm_ = [1000 * 24.45 * 0.09458/(54.1 * 0.136 * 1^1.84^ * 1.44)] = 218.2627
Partial time-weighted average (ppm) if in the plume:	TWA (task 305) = [E_ppm_(LL) * duration (LL) * frequency (LL)]/480 = [218.2627 * 10 * 4]/480 = 18.1886 ppm
Upper limit (UL):	
Point source exposure if operator was in the emission plume:	E_ppm_ = [1000 * 24.45 * 0.141875 /(54.1 * 0.136 * 1^1.84^ * 0.42)] = 1122.5334
Partial time-weighted average (ppm) if in the plume:	TWA (task 305) = [E_ppm_(UL) * duration (UL) * frequency (UL)]/480 = [1122.5334 * 20 * 4]/480 = 187.0889 ppm

**Table 5A.** Distribution of butadiene (BD) ppm-minutes, 1943–1983, task 305, plant four.

**Description** We computed an approximate probability distribution of the butadiene exposure intensity for task 305 by assuming that each parameter in the exposure model followed a triangular distribution with a mode at the midpoint between the lower and upper boundaries, by identifying the 1^st^, 2^nd^, …, 99^th^ percentile of this distribution, and by computing the exposure intensity for all possible combinations of parameter quantiles (i.e., for the approximate joint distribution of the exposure parameters). We evaluated the resulting empirical distribution of exposure estimates to find the approximate 1^st^, 2^nd^, …, 99^th^ percentile of each task-and time period-specific exposure intensity estimate.					
**Results**					

**Percentile of probability distribution**	**BD ppm-minutes**	**Percentile of probability distribution**	**BD ppm-minutes**	**Percentile of probability distribution**	**BD ppm-minutes**

1–80	0	87	21850.47	94	28984.96
81	5866.33	88	22790.27	95	30450.26
82	12203.21	89	23681.70	96	32126.42
83	17268.85	90	24669.88	97	34454.32
84	18717.13	91	25609.69	98	37932.57
85	19896.17	92	26594.45	99	45111.79
86	20910.66	93	27721.00		

#### III. Derivation of Butadiene PPM Estimates for Work Area/Job Group 817 from Component Tasks

We combined the approximate probability distributions of the five component tasks to obtain the final approximate probability distribution of the eight-hour time weighted average exposure intensity for work area/job group 817 (Table 6A). To do this, we: 1) selected 100 points from each of the approximate probability distributions of exposure intensity of the first two component tasks, each corresponding to the mid-point of the range of ppm-minutes of exposure comprised by each percentile category; 2) created a new distribution of every possible combination of these exposure intensities (100 × 100 = 10,000 possible combinations); 3) computed the sum of ppm-minutes of exposure for each combination; and 4) sorted the 10,000 resulting sums from the lowest to the highest. From that distribution we selected 100 new points of the approximate probability distribution of ppm-minutes of exposure attributable to the first two component tasks. We then combined those values with 100 selected points of the approximate probability distribution of the third component task, created a distribution of all possible combinations of exposure intensities, and selected 100 new percentile points of the exposure intensity attributed to the first three tasks. We repeated this process for each of the remaining two component tasks of work area/job group 817. This procedure, at its completion yielded a dataset with 10,000 observations corresponding to the approximate probability distribution of the ppm-minutes of butadiene exposure for work area/job group 817 during a specified calendar year. The arithmetic mean of this distribution, divided by 480 minutes in a work shift, is the butadiene ppm value that we used as the eight-hour TWA of butadiene exposure in the main analysis.

We used the entire percentile distribution of butadiene TWAs created for work area/job groups, to create the 1,000 uncertainty analysis datasets. To obtain each dataset, we randomly selected a percentile for each work area/job group in a particular plant and chose for that work area/job group the set of butadiene ppm values corresponding to that percentile (i.e., for a given work area/job group in a given plant, we used the same percentile *for all years* to select butadiene ppm values). We compiled 1,000 JEMs containing butadiene ppm values selected according to each set of randomly selected percentiles, linked the 1,000 JEMs to subjects’ work histories, and recalculated all subjects’ cumulative exposure to butadiene for each iteration. Table 7A displays the work history and exposure estimates of our sample subject from the 70^th^ of 1,000 uncertainty analysis datasets. In dataset 70 of the uncertainty analysis, the butadiene ppm eight-hour TWA for work area/job group 817 in 1950 was based on the 25^th^ percentile of the approximate probability distribution of exposure intensities for work area/job group 817.

**Table 6A.** Combination of task-specific butadiene exposure estimates to obtain the distribution of eight-hour time-weighted average estimates (BD ppm) for work area/job group 817, plant four, 1950.

**Procedure** Using the five component tasks for work area/job group 817 that entailed butadiene exposure, we computed the approximate probability distribution of the eight-hour time weighted average exposure intensity. We selected 100 points from each of the approximate probability distributions of exposure intensity of the first two component tasks, and created a new distribution of every possible combination of these exposure intensities (100 * 100 = 10,000 possible combinations). From that distribution we selected 100 new points of the approximate probability distribution of exposure intensity attributable to the first two component tasks. We then combined those values with 100 selected points of the approximate probability distribution of exposure intensity of the third component task, created a distribution of all possible combinations of exposure intensities, and selected 100 new percentile points of the exposure intensity attributed to the first three tasks. We repeated this process for each of the other two component tasks of work area/job group 817. **Distribution of estimates for work area/job group 817** Below are selected values of the approximate probability distribution of BD ppm-minutes for work area/job group 817 in plant four in 1950.					
**Percentile of probability distribution**	**BD ppm-minutes**	**Percentile of probability distribution**	**BD ppm-minutes**	**Percentile of probability distribution**	**BD ppm-minutes**

5	9213.70	45	14415.39	85	33112.75
10	10208.70	50	15007.26	90	38563.94
15	10920.08	55	15666.86	95	45231.64
20	11557.11	60	16382.51		
25	12149.74	65	17295.72	min	5435.93
30	12718.85	70	18323.38	mean	20699.60
35	13283.74	75	19912.71	max	130474.33
40	13788.17	80	23472.45		

**Calculation of BD ppm 8-hour TWA for work area/job group 817, plant four, 1950 (main analysis)** BD ppm 8-hour TWA = mean of approximate probability distribution/ 480 minutes = 20699.60/480 = 43.1242					
**Calculation of BD ppm 8-hour TWA for work area/job group 817, plant four, 1950 (dataset 70 of uncertainty analysis)** In the 70^th^ of the 1,000 datasets created for the uncertainty analysis, we randomly selected the 25^th^ percentile of approximate probability distribution of butadiene exposure intensity for work area/job group 817 in plant four. BD ppm 8-hour TWA = 25^th^ percentile value of approximate probability distribution/ 480 minutes = 12149.74/480 = 25.3120					

**Table 7A.** Work history of one subject with butadiene (BD) ppm used in dataset 70 of uncertainty analysis.

**Segment**	**Work area/job group**	**Start date**	**End date**	**Days**	**BD ppm 8-hour TWA**	**BD ppm-years**
1	812	08/27/1943	09/24/1943	28	18.5570	1.42
2	812	09/24/1943	12/31/1943	98	18.5570	6.40
3	812	12/31/1943	01/23/1944	23	18.5622	7.57
4	812	01/23/1944	10/01/1944	252	18.5622	20.38
5	817	10/01/1944	12/31/1944	91	25.3114	26.68
6	816	12/31/1944	12/12/1945	346	16.4205	42.24
7	816	12/12/1945	12/31/1945	19	16.4205	43.09
8	816	12/31/1945	01/06/1946	6	16.4347	43.36
9	816	01/06/1946	12/31/1946	359	16.4347	59.52
10	816	12/31/1946	12/31/1947	365	16.4499	75.96
11	816	12/31/1947	10/31/1948	305	16.6307	89.84
12	817	10/31/1948	12/31/1948	61	25.3126	94.07
13	817	12/31/1948	12/31/1949	365	25.3045	119.36
14	817	12/31/1949	10/22/1950	295	25.3120	139.80
15	817	10/22/1950	12/31/1950	70	25.3120	144.65
16	817	12/31/1950	12/31/1951	365	25.3614	169.20
17	817	12/31/1951	12/31/1952	366	25.3120	195.36
18	817	12/31/1952	12/31/1953	365	25.3366	220.68
19	817	12/31/1953	12/31/1954	365	25.3100	245.97
20	817	12/31/1954	12/31/1955	365	25.3139	271.27
21	817	12/31/1955	12/31/1956	366	25.3084	296.63
22	817	12/31/1956	12/31/1957	365	25.3092	321.92
23	817	12/31/1957	12/31/1958	365	25.3137	347.22
24	817	12/31/1958	12/31/1959	365	25.3126	372.51
25	817	12/31/1959	12/31/1960	366	23.7432	396.30
26	817	12/31/1960	12/31/1961	365	23.7102	415.00
27	817	12/31/1961	12/31/1962	365	23.7005	443.68
28	817	12/31/1962	03/14/1963	74	23.6767	448.41
29	817	04/30/1963	12/31/1963	245	23.6767	464.30
30	817	12/31/1963	12/31/1964	366	23.7113	488.06
31	817	12/31/1964	12/31/1965	365	23.6885	511.73
32	817	12/31/1965	12/31/1966	365	23.6767	535.39
33	817	12/31/1966	12/31/1967	365	23.6888	559.06
34	817	12/31/1967	12/31/1968	366	23.6718	582.78
35	817	12/31/1968	10/31/1969	304	23.7432	602.54
36	817	10/31/1969	12/31/1969	61	23.7432	606.51
37	817	12/31/1969	12/31/1970	365	23.6718	630.16
38	817	12/31/1970	04/29/1971	120	23.7115	637.89

* Work area/job group 812 = polymerization operative, unspecified; 816 = polymerization reactor recovery; 817 = recovery in SBR polymerization.
